# Hierarchy of evidence relating to hand surgery in Brazilian orthopedic journals

**DOI:** 10.1590/S1516-31802011000200007

**Published:** 2011-03-03

**Authors:** Vinícius Ynoe de Moraes, João Carlos Belloti, Fábio Ynoe de Moraes, José Antonio Galbiatti, Evandro Pereira Palácio, João Baptista Gomes dos Santos, Flávio Faloppa

**Affiliations:** I MD. Orthopedic Surgeon, Universidade Federal de São Paulo — Escola Paulista de Medicina (Unifesp-EPM), São Paulo, Brazil.; II MD, PhD. Professor in the Department of Orthopedics and Traumatology, Universidade Federal de São Paulo (Unifesp), São Paulo, Brazil.; III Medical student, Faculdade Estadual de Medicina de Marília (Famema), Marília, São Paulo, Brazil.; IV MD, PhD. Professor in the Department of Orthopedics and Traumatology, Faculdade Estadual de Medicina de Marília (Famema), Marília, São Paulo, Brazil.; V MD. Orthopedic Surgeon, Faculdade Estadual de Medicina de Marília (Famema), Marília, São Paulo, Brazil.; VI MD, PhD. Chairman of Orthopedics and Traumatology, Universidade Federal de São Paulo (Unifesp), São Paulo, Brazil.

**Keywords:** Orthopedics, Hand, Epidemiologic methods, Research design, Evidence-based medicine, Ortopedia, Mãos, Métodos epidemiológicos, Projetos de pesquisa, Medicina baseada em evidências

## Abstract

**CONTEXT AND OBJECTIVE:**

There is no systematic assessment of the quality of scientific production in the specialty of hand surgery in our setting. This study aimed to systematically assess the status of evidence generation relating to hand surgery and to evaluate the reproducibility of the classification method based on an evidence pyramid.

**DESIGN AND SETTING:**

Secondary study conducted at Universidade Federal de São Paulo (Unifesp) and Faculdade Estadual de Medicina de Marília (Famema).

**METHODS:**

Two researchers independently conducted an electronic database search for hand surgery studies published between 2000 and 2009 in the two main Brazilian orthopedic journals (Acta Ortopédica Brasileira and Revista Brasileira de Ortopedia). The studies identified were subsequently classified according to methodological design (systematic review of the literature, randomized clinical trial, cohort study, case-control study, case series and other studies) and evidence level (I to V).

**RESULTS:**

A total of 1,150 articles were evaluated, and 83 (7.2%) were included in the final analysis. Studies with evidence level IV (case series) accounted for 41 (49%) of the published papers. Studies with evidence level V (other studies) accounted for 12 (14.5%) of the papers. Only two studies (2.4%) were ranked as level I or II. The inter-rater reproducibility was excellent (k = 0.94).

**CONCLUSIONS:**

Hand surgery articles corresponded to less than one tenth of Brazilian orthopedic production. Studies with evidence level IV were the commonest type. The reproducibility of the classification stratified by evidence level was almost perfect.

## Abstract (pt)

**CONTEXTO E OBJETIVO:**

Não há uma avaliação sistemática da qualidade da produção científica na especialidade de cirurgia da mão em nosso meio. Este estudo objetiva analisar o status da geração de evidências em cirurgia da mão, por meio de avaliação sistematizada, além de avaliar a reprodutibilidade da classificação baseada na pirâmide das evidências.

**TIPO DE ESTUDO E LOCAL:**

Estudo secundário realizado pela Universidade Federal de São Paulo (Unifesp) e Faculdade Estadual de Medicina de Marília (Famema).

**MÉTODOS:**

De forma independente, dois pesquisadores promoveram uma busca eletrônica dos trabalhos envolvendo cirurgia da mão, no período de 2000–2009, para duas principais revistas na literatura ortopédica nacional (Acta Ortopédica Brasileira e Revista Brasileira de Ortopedia). Os trabalhos identificados foram subsequentemente classificados pelo tipo de delineamento metodológico (revisões sistemáticas da literatura, estudos clínicos randomizados, estudos de coorte, estudos caso-controle, séries de casos e outros estudos) e nível de evidência (I a V).

**RESULTADOS:**

Foram avaliados 1.150 artigos, sendo 83 (7,2%) considerados para a análise final. Estudos com nível de evidência IV (séries de caso) correspondem a 41 (49%) das publicações. Estudos nível de evidência V (outros estudos), corresponderam a 12 (14,5%) da amostra. Apenas dois estudos (2,4%) foram identificados como nível I ou II. A reprodutibilidade interobservadores foi excelente (k = 0.94).

**CONCLUSÕES:**

Os artigos de cirurgia da mão correspondem a menos de um décimo da produção ortopédica nacional. Estudos nível de evidência IV foram os mais encontrados na literatura nacional. A classificação estratificada por níveis de evidência apresenta reprodutibilidade quase perfeita.

## INTRODUCTION

The notion of evidence-based medicine (EBM) was introduced in 1991 and has been increasingly accepted by the international scientific community.^1^ In 2003, prominent journals began to use evidence hierarchies to rank the published studies. As a result, EBM concepts were adopted by the main specialty conferences and symposia.^2,3^ The importance and acceptance of the EBM concepts were measured in an article published in the British Medical Journal (BMJ) in 2007, in which the editors described the emergence of EBM as one of the 15 most important milestones for progress in the practice of medicine since the founding of BMJ (1870) along with other milestones such as the discoveries of antimicrobials, anesthetics and DNA.^4,5^

Since then, critical analysis of the orthopedic literature has become indispensable for orthopedists and hand surgeons who require evidence from studies of good methodological quality. In addition, many steps have been taken to disseminate the EBM concepts that apply to the particular characteristics of orthopedics,^6,7^ along with critical appraisal of the methodological quality of published studies.^3,8-13^

In accordance with this paradigm, efforts have been undertaken within hand surgery to follow the EBM movement.^14–16^ However, there is a lack of data on the quality of the evidence produced to date, particularly with regard to literature published within specific countries.

## OBJECTIVES

This study aimed to: identify hand surgery studies published over the last decade (2000-2009) in the Brazilian orthopedics journals Acta Ortopédica Brasileira (AOB) and Revista Brasileira de Ortopedia (RBO); classify the types of study and evidence levels according to evidence-based medicine criteria; and observe the inter-rater agreement in the classification of the studies included.

## METHODS

Using an electronic database, two researchers independently evaluated all studies published in all editions of the AOB and RBO between 2000 and 2009. These two journals were chosen because they were national journals with an orthopedics scope and journals that were indexed in at least one international bibliographic database. The studies were initially screened based on their titles and were classified as eligible, potentially eligible and not eligible. The initial inclusion criteria included the presence of the following themes in the titles: hand and wrist fractures; peripheral nerve lesions and vascular lesions in the upper limbs; nail bed lesions; brachial plexus lesions; muscle tendon lesions; upper limb skin coverage; microsurgery; upper limb pain syndromes; upper limb congenital malformations; and anatomical and experimental studies. After this initial screening, the eligible and potentially eligible studies were again screened: first using the abstracts and then, the full-text articles. Any disagreements were resolved by a third evaluator.

After screening, the studies were assessed by the two examiners, who subsequently categorized them in terms of study type and study evidence level, in accordance with a widely used classification method that has been adapted to the orthopedic literature.^3,17^ The categorization was conducted after reading the full texts of the eligible studies. We took systematic reviews of randomized clinical trials to represent evidence level I; randomized clinical trials, level II; cohort and case-control studies, level III; case series, level IV; and narrative reviews and other designs, level V.^18,19^

For all the studies ultimately included, we obtained information regarding the journal (AOB or RBO); geographic location of the study (South, Southeast, or North-Northeast-Midwest); strength of the effect estimate in the study;^20^ and publication period (2000–2004 or 2005–2009). Case reports and studies that were not primarily clinical studies (e.g. biomechanical, anatomical, histological and molecular studies) were classified as "other studies".

### Statistical analysis

The assumption of normal distribution in the sample was assessed using the Kolmogorov-Smirnov test. Cohen's kappa test was used to assess reliability and evaluate the internal consistency of the inter-rater classifications. The interpretation of the agreement magnitude was performed based on the proposal of Landis and Koch:^21^ I. < 0, representing no agreement; II. 0 to 0.20, slight agreement; III. 0.21 to 0.40, fair agreement; IV. 0.41 to 0.60, moderate agreement; V. 0.61 to 0.80, significant agreement; and VI. 0.81 to 1.00, almost perfect agreement. The chi-square test was used to evaluate the proportions between the two periods. We considered P-values < 0.05 to be statistically significant.

## RESULTS

**Graph 1:**
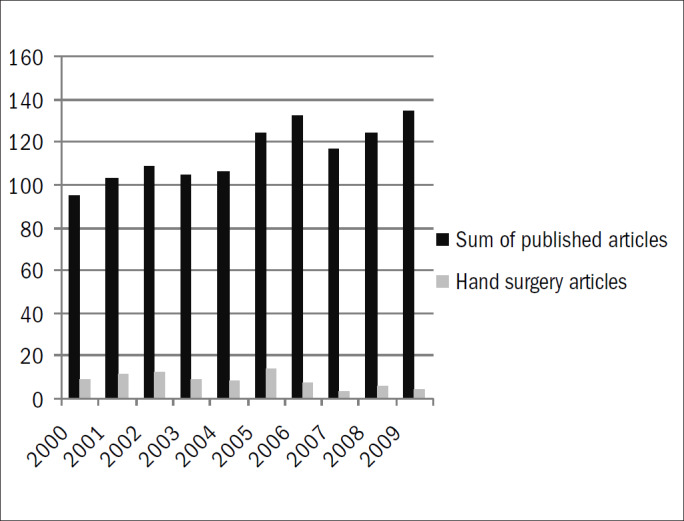
Numbers of papers published by the journals Acta Ortopédica Brasileira and Revista Brasileira de Ortopedia, distributed according to year of publication.

**Graph 2:**
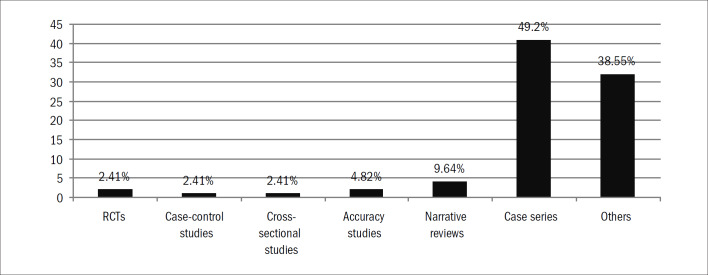
Distribution of studies according to study design. RCT=randomized controlled trial.

**Graph 3:**
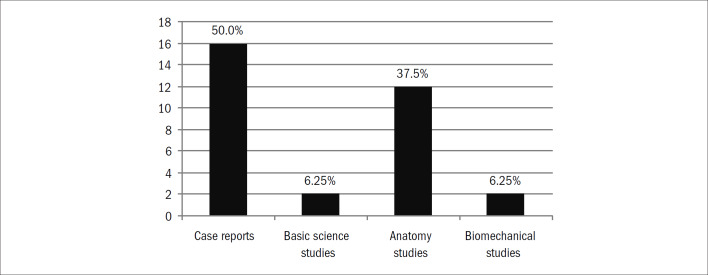
Distribution of study group "others".

We identified 1,150 studies. Of these, 83 (7.2%) were considered eligible for this present study (Graph 1; Appendix 1). Over half of these studies from both journals were case series (Table 1; Graph 2). The AOB (Table 2) published 21 (25%) of the studies (Table 2). The inter-rater reliability for the classification of study type according to the kappa statistic demonstrated almost perfect agreement (kappa = 0.94). The proportions of hand surgery studies grouped according to journal showed a statistically significant difference, such that a larger proportion of these studies were published in the RBO (Table 2; chi-square test, P = 0.02). The total number of hand surgery papers published decreased between 2000–2004 and 2004–2009 (Table 3; chi-square test, P = 0.01). Among the studies classified as "other," the majority were individual case reports (Graph 3).

**Table 1.** Frequencies of studies grouped according to the period, journal and region of origin

Study type		Clinical trial	Accuracy study	Case-control study	Cross-sectional study	Case-series	Narrative review
		n (%)	n (%)	n (%)	n (%)	n (%)	n (%)
Period	2000-2004	1 (2.9%)	1 (2.0%)	0	1 (2.0%)	28 (57.1%)	3 (6.1%)
	2005-2009	1 (2.9%)	1 (2.9%)	1 (2.9%)	0	13 (38.2%)	1 (2.9%)
Journal	AOB	2 (9.5%)	1 (4.8%)	0	0	12 (57.1%)	2 (9.5%)
	RBO	0	1 (1.6%)	1 (1.6%)	1 (1.6%)	29 (46.8%)	2 (3.2%)
Region of origin	North-Northeast-Midwest	0	0	0	0	4 (57.1%)	0
	International	0	0	0	0	0	0
	Southeast	2 (3.4%)	2 (3.4%)	1 (1.7%)	1 (1.7%)	28 (47.5%)	4 (6.8%)
	South	0	0	0	0	9 (60.0%)	0

AOB = Acta Ortopédica Brasileira; RBO = Revista Brasileira de Ortopedia.

**Table 2.** Distribution of studies on hand surgery and total number of studies published, categorized by period of publication

Period	Hand	Total
2000-2004	49 (9.4%)	518
2005-2009	34 (5.3%)	632
**Total**		**1150**

Chi-square test, P = 0.01

**Table 3.** Distribution of studies on hand surgery and total number of studies published, categorized according to the journal of publication

Journal	Hand	Total
RBO	62 (8.6%)	714
AOB	21 (4.8%)	436
**Total**		**1150**

Chi-square test, P = 0.02. AOB = Acta Ortopédica Brasileira; RBO = Revista Brasileira de Ortopedia.

## DISCUSSION

This analysis on the available literature demonstrated that a large number of hand surgery studies, particularly the case series, presented a low level of methodological evidence. High-impact orthopedic journals have also been shown to be populated with studies presenting low levels of methodological evidence.^9^ The high prevalence of published case series suggests the following hypotheses: these studies are low-cost studies that require little planning and prior knowledge; and they may be part of the routine of a healthcare group.

Therefore, the published studies were conducted as a byproduct of healthcare processes and without the goal of scientific research per se. These practices may be attributed to the lack of a scientific research tradition that promotes adequate methodological quality. Regional aspects appear to be a factor; most of the Brazilian scientific research is concentrated in the South-Southeast axis.

To place our results within the context of other orthopedic journals, we note that a study^22^ that evaluated articles published in the Journal of Bone and Joint Surgery (an American journal) found a large proportion of evidence level I and II published papers (36%), and that this proportion had increased over the preceding 30 years ago, from a proportion of 9%. Our study found that the frequency of level I and II published papers was 3%, thus suggesting the following hypotheses: 1) Higher-level studies may be more likely to be published in the most widely-indexed international journals; and 2) There is a real deficit of studies of higher methodological quality in Brazil.

The low proportion of hand surgery studies reveals a need to stimulate more scientific research in hand surgery centers with a focus on the pursuit of better evidence. The large numbers of case series and anatomical and biomechanical studies demonstrate that the search for new knowledge is intermittent and focused on surgical practice. It is worrisome that the proportion of studies published over recent years has decreased. However, this finding may demonstrate some degree of scientific maturity among the community regarding studies of higher methodological quality, which take a longer time to complete and publish.

One limitation of the present study is the possibility that hand surgery articles are not well represented in the two journals chosen for this investigation, because other Brazilian journals that were not included in the electronic search may also have published articles on hand surgery. In addition, we did not search the international literature for Brazilian authors; such a search might lead to a more optimistic outlook regarding the proportion of high-quality studies. A previous evaluation on the reliability of the study type classification demonstrated that the evidence pyramid developed by Sackett et al.^19^ seemed to be a feasible instrument for evaluating studies, with adequate external validity.

## CONCLUSIONS

Hand surgery studies comprise less than one-tenth of Brazilian orthopedic research publish between 2000 and 2009 Among these studies, half of them present evidence level IV (case series). In our analysis, the inter-rater reliability of the evidence level classification was almost perfect.
